# TrkB-Targeted Therapy for Mucoepidermoid Carcinoma

**DOI:** 10.3390/biomedicines8120531

**Published:** 2020-11-24

**Authors:** Vivian P. Wagner, Manoela D. Martins, Esra Amoura, Virgilio G. Zanella, Rafael Roesler, Caroline B. de Farias, Colin D. Bingle, Pablo A. Vargas, Lynne Bingle

**Affiliations:** 1 Department of Oral Diagnosis, Piracicaba Dental School, University of Campinas, Piracicaba 13414-903, Brazil; manomartins@gmail.com (M.D.M.); pavargas@unicamp.br (P.A.V.); 2 Oral and Maxillofacial Pathology, Department of Clinical Dentistry, University of Sheffield, Sheffield S10 2TA, UK; ebamoura2@sheffield.ac.uk (E.A.); l.bingle@sheffield.ac.uk (L.B.); 3 Department of Pathology, School of Dentistry, Federal University of Rio Grande do Sul, Porto Alegre 90035-003, Brazil; drvirgilioccp@gmail.com; 4 Head and Neck Surgery Department, Santa Rita Hospital, Santa Casa de Misericórdia de Porto Alegre, Porto Alegre 90020-090, Brazil; 5 Cancer and Neurobiology Laboratory, Experimental Research Center, Porto Alegre Clinical Hospital, Federal University of Rio Grande do Sul, Porto Alegre 90035-903, Brazil; rafaelroesler@hcpa.edu.br (R.R.); carolbfarias@gmail.com (C.B.d.F.); 6 Department of Pharmacology, Institute for Basic Health Sciences, Federal University of Rio Grande do Sul, Porto Alegre 90050-170, Brazil; 7 Rafael Koff Acordi Research Center, Children’s Cancer Institute, Porto Alegre 90620-110, Brazil; 8 Academic Unit of Respiratory Medicine, Department of Infection, Immunity and Cardiovascular Disease, University of Sheffield, Sheffield S10 2RX, UK; c.d.bingle@sheffield.ac.uk

**Keywords:** head and neck neoplasms, salivary gland neoplasms, adenocarcinoma, cell biology, therapeutics

## Abstract

The brain-derived neurotrophic factor (BDNF)/tyrosine receptor kinase B (TrkB) pathway was previously associated with key oncogenic outcomes in a number of adenocarcinomas. The aim of our study was to determine the role of this pathway in mucoepidermoid carcinoma (MEC). Three MEC cell lines (UM-HMC-2, H253 and H292) were exposed to Cisplatin, the TrkB inhibitor, ANA-12 and a combination of these drugs. Ultrastructural changes were assessed through transmission electron microscopy; scratch and Transwell assays were used to assess migration and invasion; and a clonogenic assay and spheroid-forming assay allowed assessment of survival and percentage of cancer stem cells (CSC). Changes in cell ultrastructure demonstrated Cisplatin cytotoxicity, while the effects of ANA-12 were less pronounced. Both drugs, used individually and in combination, delayed MEC cell migration, invasion and survival. ANA-12 significantly reduced the number of CSC, but the Cisplatin effect was greater, almost eliminating this cell population in all MEC cell lines. Interestingly, the spheroid forming capacity recovered, following the combination therapy, as compared to Cisplatin alone. Our studies allowed us to conclude that the TrkB inhibition, efficiently impaired MEC cell migration, invasion and survival in vitro, however, the decrease in CSC number, following the combined treatment of ANA-12 and Cisplatin, was less than that seen with Cisplatin alone; this represents a limiting factor.

## 1. Introduction

The latest report by the International Agency for Research on Cancer (IARC) released in 2018 estimated an increase of more than 55% in new cases of salivary gland cancer (SGC), between 2018 and 2040, reaching an annual global incidence of 82,039 [1]. SGC comprise a group of more than 20 types of malignancies, recognized as a major challenge to pathologists and clinicians, due to the vast heterogeneous microscopic appearance, combined with mixed clinical behaviour [2]. Mucoepidermoid carcinoma (MEC) represents the most prevalent type of SGC [3,4]. This subtype of adenocarcinoma can also occur as a primary tumour in other sites, recognized as the most common salivary gland type carcinoma in the lungs [5]. The clinical stage at diagnosis directly influences the 5-year disease free survival rate, which in the presence of nodal or distant metastasis, can be as low as 36% and 25%, respectively [6]. Tumour grade also has an important role in prognosis, as survival rates from high-grade tumors are 43% lower than low/intermediate cases [6]. The mainstay treatment for MEC remains surgical resection, as no significant improvement was observed with non-surgical therapeutic options, for advanced disease [7].

Exceptionally, late recurrence presents an important challenge for clinicians dealing with MEC, and other SGC. Around 17% of MEC patients will present with disease relapse, 5 years after initial treatment, with higher chances of distant rather than local metastasis [8]. The ability of MEC cells to invade and migrate, resist conventional treatment and remain quiescent for long periods of time, were suggested as the main causes for unfavorable prognosis, after an early period of apparent cure. The sub-population of resilient and highly tumourigenic cells, known as cancer stem cells (CSC), might also be important in this process [9]. CSC are capable of self-renewal and multi-lineage differentiation, along with an ability to evade conventional treatments. Therefore, CSC are recognized as the key initiators of disease relapse. Emerging data demonstrated the presence of CSC in MEC and the capacity of these cells to initiate new tumors and evade conventional therapy [9,10,11]. In this scenario, new systemic therapies for MEC, able to target tumourigenic behaviours such as cell invasion and migration, combined with CSC disruption, are urgently needed.

Although genetic events are acknowledged as the main cause of tumour initiation, growth factors (GF) represent the major regulators of the successive crucial events for tumour progression, such as clonal expansion, tumour-related angiogenesis, epithelial–mesenchymal transition, besides the ability of tumour cells to evade therapies [12]. GF and their downstream pathways represent a promising area for systemic therapy, if they can be targeted by highly specific drugs, such as tyrosine kinase inhibitors (TKIs) and monoclonal antibodies (mAbs), which are usually associated with more tolerable doses and relatively mild adverse effects [12]. The brain-derived neurotrophic factor (BDNF) is a GF from the neurotrophin family, which specifically binds to the tyrosine receptor kinase B (TrkB) [13]. The BDNF/TrkB pathway activates downstream pathways such as PI3K/Akt, and is associated with key oncogenic outcomes in a variety of adenocarcinomas, including lung [14], breast [15], gastric [16], colorectal [17] and salivary gland adenoid cystic carcinoma [18,19]. Therefore, TrkB inhibition is increasingly studied as a promising therapeutic target, however, the role of the BDNF/TrkB pathway in MEC remains to be elucidated. The aim of this study was to discover the effects of the BDNF/TrkB pathway in MEC, by evaluating the outcomes of TrkB inhibition on MEC cell invasion, migration, survival and the percentage of remaining CSCs.

## 2. Experimental Section

### 2.1. Human Tissue Specimens

Cases diagnosed as MEC were retrieved from archives of the Pathology service at the Santa Rita Hospital of the Santa Casa de Misericórdia, Porto Alegre, Brazil (Human Research Ethics Committee approval: 2.324.138). Forty-one cases of MEC were included. Two experienced pathologists (MDM and PAV) reviewed the original haematoxylin–eosin-stained slides to confirm diagnosis and tumour grade, resulting in 26 low-grade, 9 intermediate-grade, and 6 high-grade cases. Follow-up information was collected from the medical records. Ten normal salivary glands (NSG) were included as controls, and were obtained as adjacent tissue from parotid (*n* = 6) and lower lip surgeries (*n* = 4), retrieved from the same pathology service.

### 2.2. Immunohistochemistry

Three micrometre sections of MEC and normal salivary glands on silanized slides were deparaffinized in xylene and hydrated in ethanol. Endogenous peroxidase activity was quenched and antigen retrieval was performed prior to incubation with primary antibodies, using 5% hydrogen peroxide and citric acid, respectively. The primary antibodies, dilutions, clone and sources were as follows: BDNF (1:750, EPR1292; Abcam, Cambridge, UK), phosphorylated-TrkB Y706 + Y070 (1:100, Polyclonal; Abcam). Diaminobenzidine tetrahydrochloride (DAB; Novocastra, Newcastle, UK) indicated positive reactions and the sections were then counterstained with Mayer’s haematoxylin. Primary antibodies were replaced with non-immune serum for negative controls. Human brain tissue was used as positive control for both antibodies. A semi-quantitative analysis was performed by assessing the percentage of positive cells at a magnification of 400×. In NSG, parenchymal cells including acinar, ductal and myoepithelial cells were analysed, while in MEC, malignant cells were assessed. A cell was considered positive when the brown, cytoplasmic staining was compatible in intensity to protein expression (and not weak background staining). Each case was classified as follows: 0—no positive cells; 1—between 1% and 25% of positive cells; 2—between 26% and 50% of positive cells; 3—between 51% and 75% of positive cells; and 4—more than 76% of positive cells. Two experienced and previously calibrated pathologists (V.P.W. and M.D.M.) performed the analysis on the basis of a consensus. All immunohistochemical reactions were performed at the same time and DAB exposure was exactly the same for all sections, however, to exclude possible misleading reaction intensity data, only the percentage of cells was assessed.

### 2.3. Cell Lines and Treatments

Three MEC cell lines, UM-HMC-2 (intermediate-grade, parotid gland), H253 (ATCC^®^ HTB-41™—undifferentiated high-grade, submandibular gland) and H292 (ATCC^®^ CRL-1848™—primary pulmonary) were used. UM-HMC-2, kindly provided by Dr. Jaques Eduardo Nör, was established at the University of Michigan School of Dentistry [20] and was cultured in DMEM-High glucose (Hyclone Laboratories Inc., Logan, UT, USA), supplemented with 10% Foetal Bovine Serum (FBS, Thermo Scientific, Waltham, MA, USA), 1% antibiotics (Invitrogen, Carlsbad, CA, USA), 1% L-glutamine (Invitrogen, Carlsbad, CA, USA), 20 ng/mL epidermal growth factor (PeproTech, Rocky Hill, NJ, USA), 400 ng/mL hydrocortisone (Sigma-Aldrich, St. Louis, MO, USA) and 5 μg/mL insulin (Sigma-Aldrich). H253 and H292 were acquired from the American Type Culture Collection (ATCC, Manassas, VA, USA) and maintained according to the ATCC prescribed guidelines. Normal salivary gland primary cells were included for comparative purposes. The primary cells were isolated from submandibular gland tissue obtained from human surgical biopsies (NREC Ethic approval number 13/NS/0120). The cells were grown in keratinocyte growth medium (KGM), which consists of DMEM-low glucose supplemented with 23% Ham’s F12 (Sigma-Aldrich), 10% Foetal bovine serum, 100 µg/mL penicillin and 100 U/mL streptomycin, 2 mM L-glutamine, 180 µM adenine (Sigma-Aldrich), 0.5 µg/mL hydrocortisone and 10 ng/mL epidermal growth factor.

The cells were grown at 37 °C with 5% CO_2_ in a standard bench-top CO_2_ incubator, monitored daily using a phase contrast microscope, and cultured to a maximum of 70% confluence before passage, to avoid stress. All experiments were carried out with a maximum of 10 passages. Cells were treated with Cisplatin (Sigma-Aldrich) and ANA-12 (Sigma-Aldrich). The IC50 of the drugs being previously established for each cell line, using a crystal violet viability assay in monolayer adhered cells [21]. For UM-HMC-2, H253 and H292 cells, the IC50 doses for Cisplatin were 15.4 µM, 13.2 µM and 14.5 µM, respectively and for ANA-12, these were 13.6 µM, 14.7 µM and 10.8 µM, respectively. All cell lines were routinely tested to confirm the absence of mycoplasma contamination.

### 2.4. PCR

MEC cells and normal primary salivary gland cells were grown to 70% confluence in normal growth media. Total RNA was isolated using an RNeasy mini kit (Qiagen, Hilden, Germany) and quantified using a NanoDrop spectrophotometer (Thermo-Fischer, Waltham, MA, USA). A high capacity cDNA reverse transcription kit (Applied Biosystems, Foster City, CA, USA) was used for synthesis of cDNA, according to the manufacturer’s instructions. Transcript levels of BDNF and TrkB were measured using SYBR Green conventional PCR (Primer sequences: BDNF—F: 5′-GGCTATGTGGAGTTGGCATT-3′ and R: 5′-CTTCAGAGGCCTTCGTTTTG-3′; TrkB—F: 5′-TGGTGCATTCCATTCACTGT-3′ and R: 5′-CGTGGTACTCCGTGTGATTG-3′). GAPDH was used as the housekeeping gene for the loading control. PCR products were visualized on 1.5% agarose gels in Tris-EDTA buffer, stained with ethidium bromide and a GeneRuler 100 bp DNA ladder (Thermo Scientific, Cheshire, UK), was used as a marker to assess product size. The band images were acquired using a Syngene Gel-Doc system with the InGenius 3 GENEsys software (Syngene, Synoptics Group, Cambridge, UK).

### 2.5. Transmission Electron Microscopy (TEM)

Cells were cultured to 70% confluence before treatment with a single dose of ANA-12, a single dose of Cisplatin or a combination of drugs in which ANA-12 was administered for 30 min, the media containing the drug removed, and then the Cisplatin administered. After 24 h, the culture medium was washed off the specimens, which were then fixed in 2.5% Glutaraldehyde/0.1 M Sodium Cacodylate buffer overnight. Excess fixative was removed the following day, by two washes of 10 min each with 0.1 M Sodium Cacodylate buffer. Samples were post-fixed in 2% aqueous Osmium Tetroxide, dehydrated through a graded series of ethanol solutions in water (50%, 75%, 95%, 100% twice and dried 100% ethanol), cleared of excess ethanol in epoxypropane (EPP) and then infiltrated in a 50/50 Araldite resin:EPP mixture, overnight on a rotor. The next day, this mixture was replaced with two changes, over 8 h of fresh Araldite resin mixture, before being embedded into EM moulds and cured in a 60 °C oven, for 48–72 h. Ultrathin sections, approximately 85 nm thick, were cut on a Leica UC6 ultra microtome onto 200 mesh copper grids. These were stained for 30 min with saturated aqueous Uranyl Acetate, washed in distilled water, and then stained in Reynold’s Lead Citrate for 5 min. The sections were examined using an FEI Tecnai Spirit Biotwin 120 Transmission Electron Microscope, at an accelerating voltage of 80 Kv. Electron micrographs were recorded using Gatan Orius 1000 digital camera and Gatan Digital Micrograph software (Gatan, Pleasanton, CA, USA). Sample fixing, embedding, and sectioning was performed by the TEM facility at the University of Sheffield.

### 2.6. Scratch Assay

MEC cells were seeded in 6-well culture dishes maintained at 37 °C and grown to confluence in normal growth media. Two hours before the scratch, the cells were treated with 2 µg/mL Mitomycin C (Sigma-Aldrich). The optimal dose of Mitomycin C was previously calculated to ensure minimal loss of viability, with maximum inhibition of cell division. After removing the media containing Mitomycin C and washing the cells with PBS, a wound was created by scratching the cells with a 200-μL pipette tip. Monotherapy groups were treated with ANA-12 or Cisplatin, while the combined group received ANA-12 for 30 min, followed by Cisplatin, as previously detailed. After 24 and 48 h, further drugs were administered, following the same protocol. Cells were allowed to migrate into the wound area until the control group had achieved complete wound closure. Photographs were taken every 8 h, using a digital camera attached to a phase contrast microscope. Experiments were performed in triplicate, and the same two areas of each well were analysed at each time point. The wound area was measured using the MRI Wound Healing Tool plugin (http://dev.mri.cnrs.fr/projects/imagej-macros/wiki/Wound_Healing_Tool) in ImageJ (National Institutes of Health, Bethesda, MD, USA), and a relative wound closure (%) was determined by normalizing the values to the wound area at T0.

### 2.7. Transwell Invasion Assay

Cell invasion through extracellular matrix (ECM) substitute was assessed using 8 µm-pore, 24-well Millicell Cell Culture Inserts (Millipore, Billerica, MA, USA), coated with fibronectin (Sigma-Aldrich). MEC cells were seeded into the upper chamber of the insert, using normal cell culture media and were allowed to adhere. After 4 h, the upper chamber media was replaced with 2% FBS media, the normal media (10% FBS) remained in the bottom chamber. The cell density and time of invasion was optimized for each cell line and the Cisplatin, ANA-12 or a combination of drugs was administered following the protocol outlined for the scratch assay. Membranes were washed with PBS and the non-invading cells were removed from the upper surface of the membrane by “scrubbing” with a cotton swab. The membranes were then fixed with methanol, stained with haematoxylin and eosin (H&E), and mounted on glass slides. The experiments were performed in triplicates and two representative images per membrane were captured using a digital camera attached to a conventional optical microscope. The number of invading cells was determined using the cell counter plugin from ImageJ. Slides were blinded before photograph capture to prevent bias. The “Invasion Index” was further calculated, and represents the ratio of the percent invasion of the treated membranes versus the percent invasion of the control membranes.

### 2.8. Clonogenic Assay

Cells were seeded at 500 cells/well in 6-well culture plates. After overnight incubation, cells were treated with a single dose of ANA-12 or Cisplatin, or both drugs, using the protocol previously described. Cells were allowed to grow for an additional 7 days before fixing the colonies with Methanol and Acetic Acid (7:1) and staining with 0.1% crystal violet. The results were assessed with a conventional optical microscope and only colonies that presented >50 cells were considered [22]. Experiments were performed in triplicate.

### 2.9. Spheroid Assay

Before cell seeding, a grid was drawn on the back of the culture plates to enable orientation during spheroid counting. A total of 3000 cells/well were seeded on 6-well Corning^®^ Costar^®^ Ultra-Low attachment plates (Merck KGaA, Darmstadt, Germany). Treatment followed the same procedure as previously described, however, media was not removed after 24 h, as the cells were grown in suspension. Spheroid formation was observed daily and on day 5, the number of spheroids was assessed through phase contrast microscopy. Spheroids growing in suspension were collected, cytospun onto glass slides (1500 rpm, 4 °C, 10 min), which were fixed with methanol for 1 min at room temperature, and then stained with H&E to assess spheroid morphology. Experiments were performed in triplicate.

### 2.10. Statistical Analysis

GraphPad Prism (GraphPad Software, San Diego, CA, USA) was used for statistical analysis. Scores from immunohistochemistry analysis were compared using non-parametric tests (Kruskal–Wallis, followed by post-hoc analysis or Mann-Whitney test). Data from in vitro assays were compared using one-way ANOVA or two-way ANOVA, followed by Tukey’s multiple comparison test. Asterisks denote statistical significance (* *p* < 0.05; ** *p* < 0.01; *** *p* < 0.001; **** *p* < 0.0001, and NS *p* > 0.05).

## 3. Results

### 3.1. BDNF/TrkB Pathway in Human Salivary Glands and Mucoepidermoid Carcinoma

**Figure 1:**
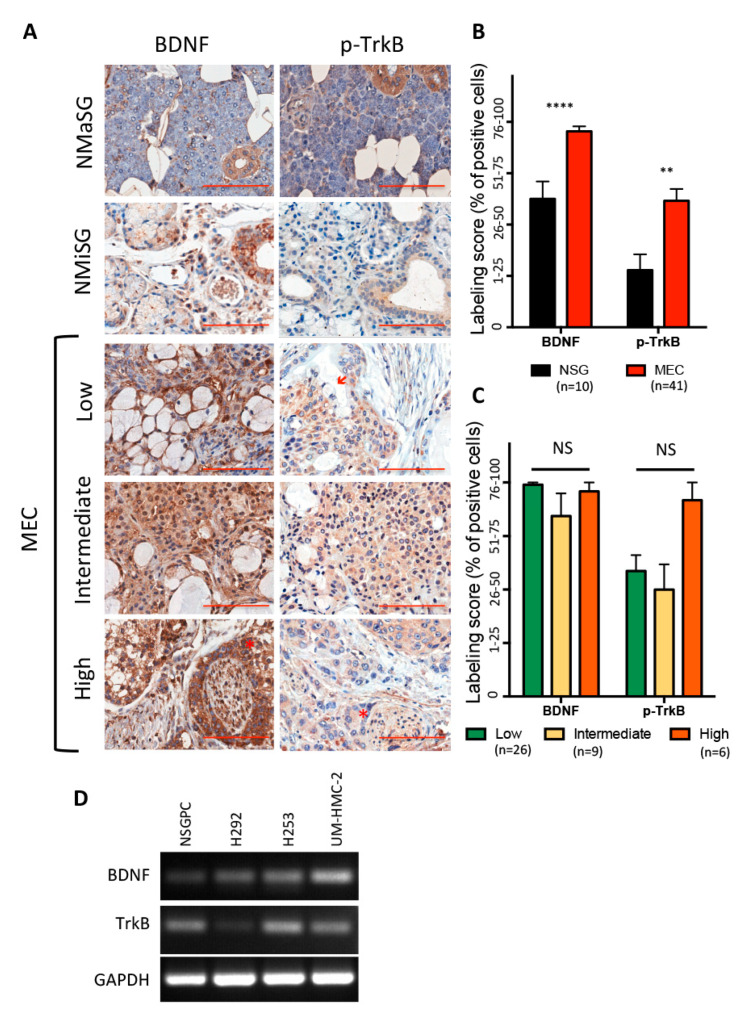
(**A**) Representative images of BDNF and pTrkB immunostaining in normal minor (MiSG) and major salivary glands (MaSG) and mucoepidermoid carcinoma (MEC) with different grades. Note the absence of pTrkB expression in mucous cells (arrow) and the presence of expression in cells associated with perineural invasion (asterisk). (**B**) The percentage of BDNF- and pTrkB-positive cells was significantly higher in MEC, compared to normal salivary gland tissue (scores obtained from the joint analysis of MaSG and MiSG) (*p* < 0.0001 and *p* = 0.0075, respectively, Mann-Whitney Test). (**C**) No significant difference was detected among the different MEC grades concerning BDNF and pTrkB expression (*p* > 0.05, Kruskal-Wallis Test), however, high-grade tumours presented a tendency for elevated percentage of pTrkB positive cells. (**D**) BDNF and pTrkB transcript levels in normal salivary gland primary cells (NSGPC) and MEC cell lines revealed higher BDNF expression in all MEC cell lines compared to NSGPC. Interestingly, high TrkB expression was observed in the high-grade salivary gland MEC (H253) with lower expression in the pulmonary MEC cell line (H292). The levels of UM-HMC-2 were similar to NSGPC (** *p* < 0.01; **** *p* < 0.0001, and NS *p* > 0.05).

BDNF plays crucial roles in normal and pathological states [13,23,24], however, few studies have assessed the levels of BDNF and TrkB in salivary gland tissue, under physiological conditions [25,26]. Four normal minor salivary glands (MiSG) from the lower lip and six parotid glands (MaSG) demonstrated BDNF and pTrkB expression, mainly in the ductal cells (Figure 1A), with a higher percentage of normal cells expressing BDNF than pTrkB, especially in MiSG.

BDNF and pTrkB expression in human MEC samples was compared to that in NSG. MEC is composed of different proportions of epidermoid, mucous, and intermediate cells with variable amounts of cyst formation. Moreover, other architectural and cytological features were variable between cases, and further determine the grade of the tumour [2]. MEC cases demonstrated a higher percentage of BDNF- and pTrkB-positive cells, compared to NSG (Figure 1B). Overall, BDNF and TrkB positivity was observed mainly in intermediate and epidermoid cells (Figure 1A). Statistical analysis revealed no difference in expression between grades (Figure 1C), however, this could be due to the limited number of high-grade cases studied (*n* = 6), as a marked tendency for TrkB overexpression was observed in high-grade tumours, compared to low and intermediate cases (Figure 1C). Tumour grade is an established prognostic marker for MEC and high-grade tumours are associated with poor survival rates [6], and thus, increased activation of signalling pathways associated with aggressiveness might be expected. Corroborating this hypothesis, BDNF and TrkB expression was detected in MEC cells associated with perineural invasion (Figure 1A), which is also recognized as a prognostic marker [6]. The average follow-up period was 39 months (ranging from 0 to 130 months) and during this time none of the patients died due to their tumour, hampering our analysis of BDNF and TrkB values as a biomarker for disease-specific survival. Four patients (9.7%) experienced local recurrence but no significant difference could be detected among BDNF and TrkB expression in patients who experienced recurrence compared to those who did not.

Basal levels of BDNF and TrkB were determined in three MEC cell lines harbouring different features, and further compared to levels of normal primary salivary gland cells (NSGPC). UM-HMC-2, H253 and H292 represent, respectively, intermediate-grade parotid gland MEC, undifferentiated high-grade submandibular gland MEC and primary pulmonary MEC [20,27]. All cells presented a consistent cobblestone epithelial-like morphology, throughout the study, and ultrastructural analysis revealed similar features, such as intracytoplasmic vacuoles, among the different cell types (Supplementary Figure S1A). All MEC cell lines and NSGPC expressed BDNF and TrkB mRNA (Figure 1D). BDNF levels were higher in all MEC cell lines, compared to NSGPC, corroborating the immunohistochemistry results with human tissue. Interestingly, the TrkB levels were more irregular among the cell lines. The high-grade MEC cell line, H253, presented the highest TrkB mRNA levels, while the pulmonary MEC cell line (H292) showed relatively low expression. TrkB levels in UM-HMC-2 were similar to NSGPC. Our results, therefore, suggest that TrkB is increased in high-grade MEC cases, again corroborating our immunohistochemistry results.

### 3.2. Effects of Cisplatin and TrkB Inhibition on Cell Ultrastructural Morphology

**Figure 2:**
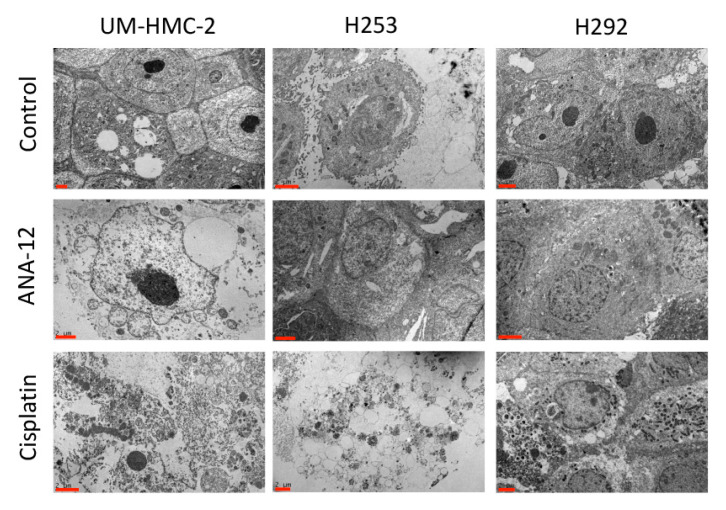
Representative images of MEC cells ultrastructural morphology following ANA-12 and cisplatin administration. Note, Cisplatin induced a high number of apoptotic changes such as decrease in cells size, “cup-shaped” chromatin condensation, convolution of the nuclear and cellular outlines, loss of cell contact, loss of specialized surface elements such as microvilli and cell–cell junctions, and ultimately cell fragmentation and formation of apoptotic bodies. Cytosolic constituents released into the extracellular space can be observed indicating a process of secondary necrosis. Scale bar—2 µm.

Platinum-based chemotherapy is the most common option for all SGCs, including MEC, however, the response rates are unsatisfactory and metastatic disease remains incurable [28,29]. Our study was designed to examine the effects of cisplatin on MEC cells in comparison to TrkB inhibition, as a new potential systemic treatment. To achieve TrkB inhibition, we used ANA-12, a low–molecular weight molecule capable of preventing TrkB activation and inhibiting downstream processes with a high potency, in a non-competitive manner, with BDNF [30]. As ANA-12 and Cisplatin differ significantly in their mechanisms of action, we analysed the effects of both drugs on MEC cell ultrastructure through transmission electron microscopy (TEM). Cisplatin is a cytotoxic drug capable of interfering with DNA repair mechanisms and causing DNA damage, subsequently triggering cell apoptosis [31]. Indeed, it was possible to identify many signs of early and late apoptosis in all MEC cell lines treated with Cisplatin, such as decrease in cell size, “cup-shaped” chromatin condensation, convolution of the nuclear and cellular outlines, loss of cell contact, loss of specialized surface elements such as microvilli and cell–cell junctions, and ultimately cell fragmentation and the formation of apoptotic bodies (Figure 2). Moreover, cytosolic constituents released into the extracellular space could be observed to indicate a process of secondary necrosis. Despite treatment with a highly cytotoxic drug, a few cells appeared healthy with normal ultrastructural morphology, suggesting intrinsic mechanisms of resistance to Cisplatin in all MEC cells lines examined. Treatment with ANA-12 also resulted in signs of apoptosis, however, this was to a lesser degree in comparison to Cisplatin. Many more cells in this group retained a healthy morphology and the apoptotic and late necrotic features were less prominent. Interestingly, no intra-cellular drug deposits were seen for either treatments, probably due to the active process of drug efflux. In our study, TEM analysis was performed 24 h after drug delivery, but a previous report demonstrated that after 4 h of treatment, most of the drug deposits were already close to the cell membrane or were outside the cell [32].

### 3.3. Effect of TrkB Inhibition and Cisplatin on MEC Cell Migration and Invasion

After establishing the effects of cisplatin and ANA-12 on ultrastructural morphology, we determined the effect of the drugs on important cell functions, mainly associated with tumour metastasis. We tested the effects of treatment with Cisplatin or ANA-12 alone and compared it to treatment with a combination of both drugs. As ANA-12 has the capacity to inhibit BDNF/TrkB downstream pathways, such as Akt, which can be associated with increased intrinsic resistance to conventional chemotherapy [33], we used this drug as a first hit, before Cisplatin, with the aim of inhibiting drug resistance.

**Figure 3:**
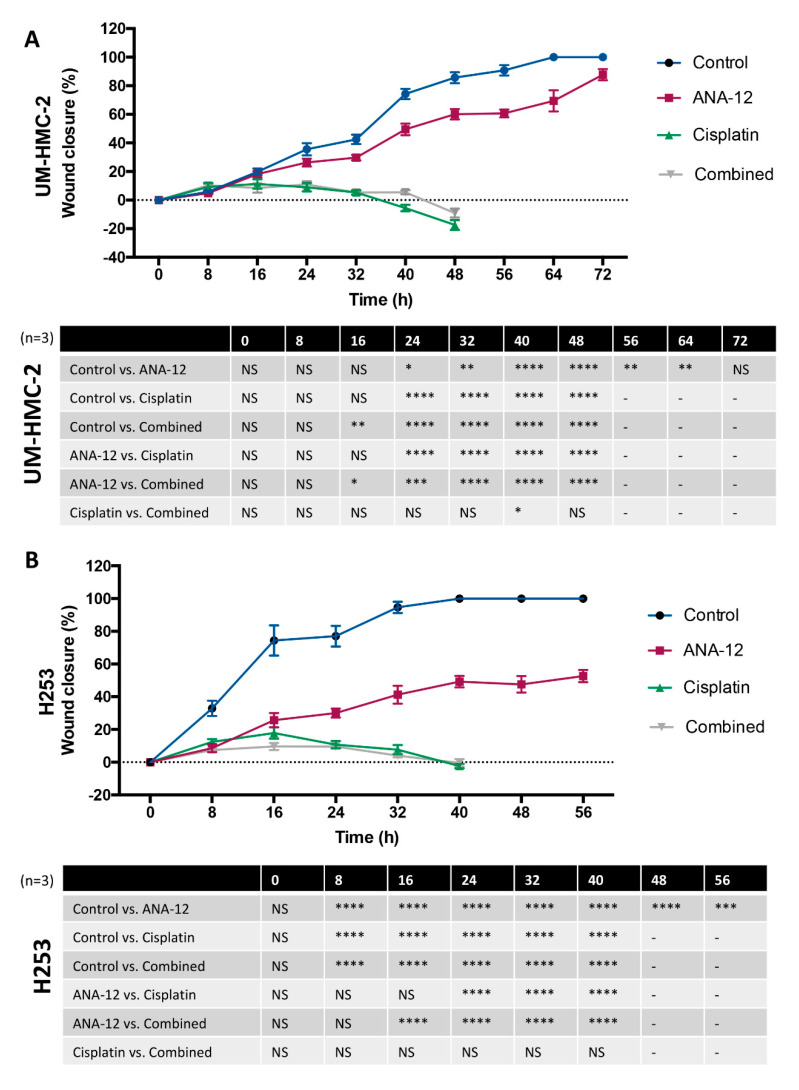
(**A**) UM-HMC-2 wound closure following ANA-12, Cisplatin and combined treatment. Note that the combined treatment presented the most promising results to delay wound closure in this cell line. (**B**) H253 wound closure following ANA-12, Cisplatin and combined treatment. Note that Cisplatin and the combined treatment results were similar and ANA-12 effects occurred earlier and in a greater magnitude, compared to UM-HMC-2 (* *p* < 0.05; ** *p* < 0.01; *** *p* < 0.001; **** *p* < 0.0001 and NS *p* > 0.05).

Cell migration was assessed using a scratch assay. To guarantee that only cell migration, and not cell proliferation, was responsible for wound closure Mitomycin C was used. MEC cells were treated with a low dose of this cell cycle arrest antagonist, before Cisplatin or ANA-12 administration. Forty-eight hours after creating the wound, no migration of lung MEC cells (H292) was observed in the control or the treated groups. This cell line was, therefore, excluded from this part of our study with only outcomes from UM-HMC-2 and H253 cells being analysed. UM-HMC-2 cells took 64 h to achieve closure of the wound in the control group, while H253 control cells showed complete wound closure after 40 h (Figure 3A,B). The faster migratory capacity of H253 might be attributed to the undifferentiated, thus more aggressive, nature of this cell line. When treated with ANA-12, time to wound closure was significantly delayed in both the UM-HMC-2 and H253 cell lines, supporting the important role of BDNF/TrkB activation during the MEC cell migration. ANA-12 significantly delayed UM-HMC-2 migratory activity from 24 to 64 h. The inhibitory effect of ANA-12 in H253 migration started earlier, just 8 h after wounding, and continued until the last evaluation time (56 h) (Figure 3A,B). This might be associated with the previously demonstrated higher mRNA expression of TrkB in H253 cells, which suggests a higher dependence of these cells on this pathway. Surprisingly, the effects of Cisplatin on the migration of both cell lines were greater than that of ANA-12. At the initial time points, cells treated with Cisplatin retained a healthy morphology and minimal migratory activity was recorded. The cytotoxicity of Cisplatin was observed at later time points, causing important changes to cell morphology and significant cell death, which completely impaired wound closure (Supplementary Figure S2A,B). Compared to ANA-12 alone, the effects of combined therapy were greater, however, ANA-12 plus Cisplatin did not differ significantly to Cisplatin-only treatment.

**Figure 4:**
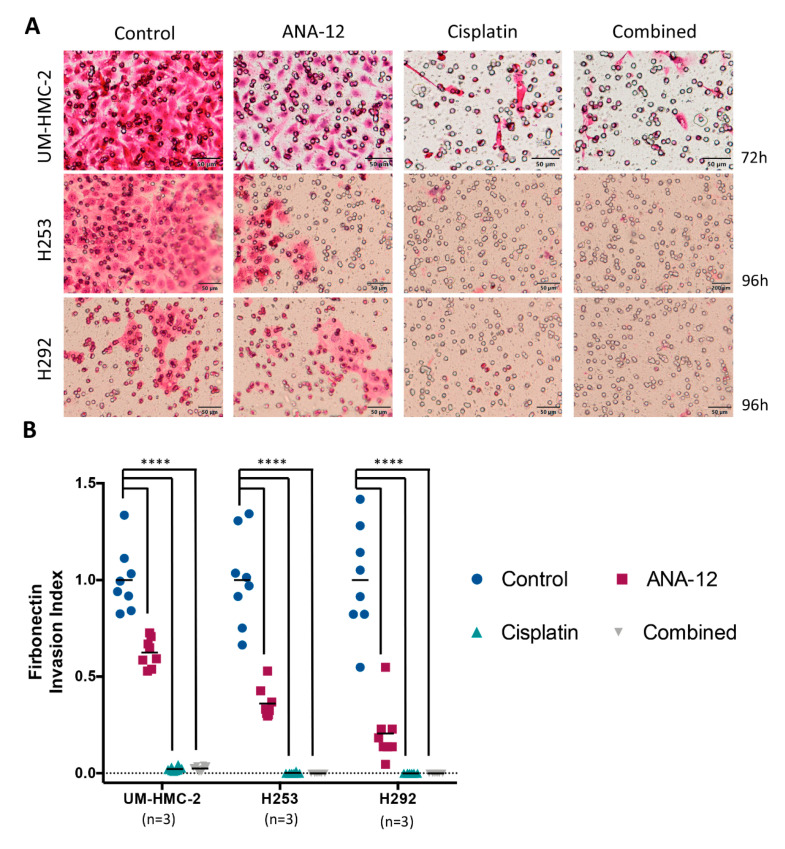
(**A**) Representative images of the invading cells following ANA-12, Cisplatin and combined treatment. Time of invasion is presented on the right side. (**B**) Quantitative analysis revealed that all treatments were effective in disrupting cell invasive capacities. Yet, note that Cisplatin and combined treatment achieved the most promising results for all cell lines (**** *p* < 0.0001).

The impact of ANA-12 and Cisplatin on cell invasion was evaluated through transwell membranes coated with fibronectin, which is abundant in the connective tissue matrix, and mimics the ECM cells needed to invade during tumour progression [34]. Optimal time to invasion was determined for each cell line. UM-HMC-2 cells invaded faster in comparison to both H253 and H292 cells. This baseline difference in invasive capacity might be due to distinctive proteinases produced by the different cells; further experiments are needed to clarify this. Treatment with ANA-12 significantly delayed cell invasion in all cell lines evaluated (Figure 4A,B). Similarly, Cisplatin treatment significantly limited the invasive capacity of all cell lines (Figure 4A,B). Recently, it was demonstrated that Cisplatin caused the accumulation of stress fibres and actin disintegration, in malignant cells in vitro, resulting in increased cell stiffness hampering the cell motility [35]. In the present study, Cisplatin was more efficient in inhibiting cell invasion than therapy targeted against TrkB. The combination of ANA-12 and cisplatin, did not increase the inhibition of cell invasion over that seen with Cisplatin alone (Figure 4A,B).

### 3.4. Effect of TrkB Inhibition and Cisplatin on MEC Cell Survival and CSC Number

We used two different approaches, clonogenic [36] and tumour spheroid assays [37], to evaluate the impact of our therapies on CSC. The number of colonies and tumour spheroids varied between the cell lines. The colony forming ability of H253 was less than that in the other cell lines, however, H253 cells formed the highest number of tumour spheroids under low-attachment conditions (Supplementary Figure S3A–C). Disparities in size and morphology of colonies and tumour spheroids were also noted. UM-HMC-2 colonies had a “looser” architecture, while H253 and H292 cells formed denser colonies. H253 and H292 also produced bigger spheres with abnormal mitotic activity and numerous cells demonstrated highly compact chromatin (Supplementary Figure S3A). Previous studies demonstrated that CSC have a deacetylated phenotype (compacted chromatin), resulting in a more undifferentiated profile through the silencing of differentiation genes, and also contributing to increased chemoresistance, due to the difficulty of compounds to access genetic material [38,39].

**Figure 5:**
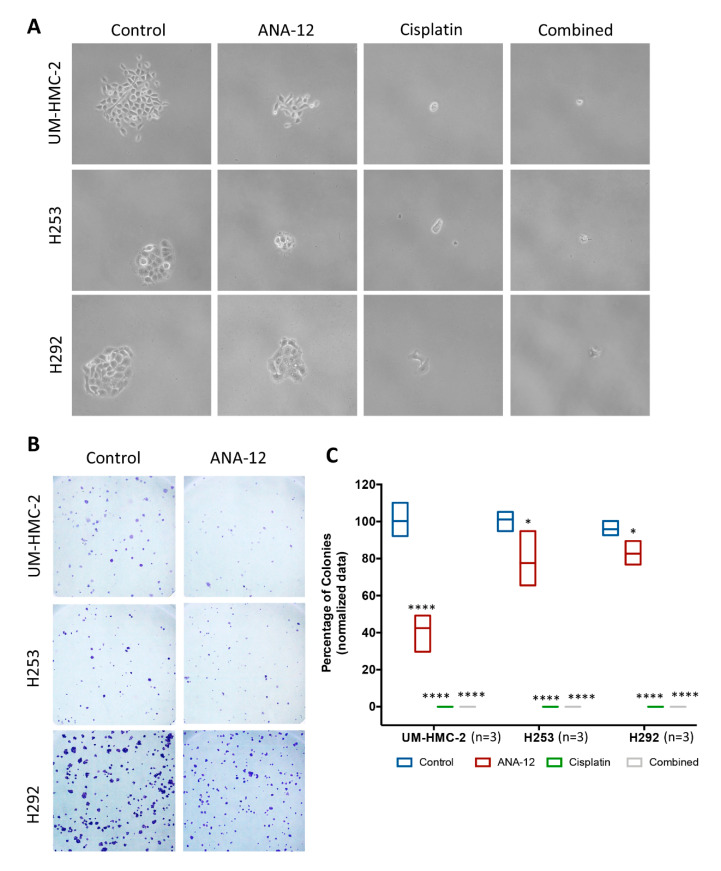
(**A**) Representative images of phase contrast microscopy during the clonogenic assay. Note that under Cisplatin and combined treatment, MEC cells remained attached and with a healthy morphology, but did not present the capacity to form colonies. (**B**) Comparison of stained colonies in control and ANA-12 groups. Note that the number and size of colonies is reduced following TrkB inhibition. (**C**) Quantitative analysis revealed a significant reduction in the number of surviving colonies (more than 50 cells) in all MEC cell lines (* *p* < 0.05; **** *p* < 0.0001).

TrkB-inhibition significantly reduced the number of surviving colonies compared to untreated cells (Figure 5A–C). ANA-12 treatment was previously associated with the cyclin-dependent kinase (CDK) inhibitor, p21, mRNA, and protein overexpression [40]. p21 acts by promoting cell cycle inhibition, thus the reduction in clonal cell expansion could be a result of p21 activation. Moreover, TrkB inhibition activates p21 at different levels, according to the cell type [40], which corroborates our results that demonstrated different magnitudes of clonogenicity inhibition in MEC cell lines. Cisplatin alone, and in combination with ANA-12, resulted in surviving isolated cells having no colony-forming capacity (Figure 5A).

**Figure 6:**
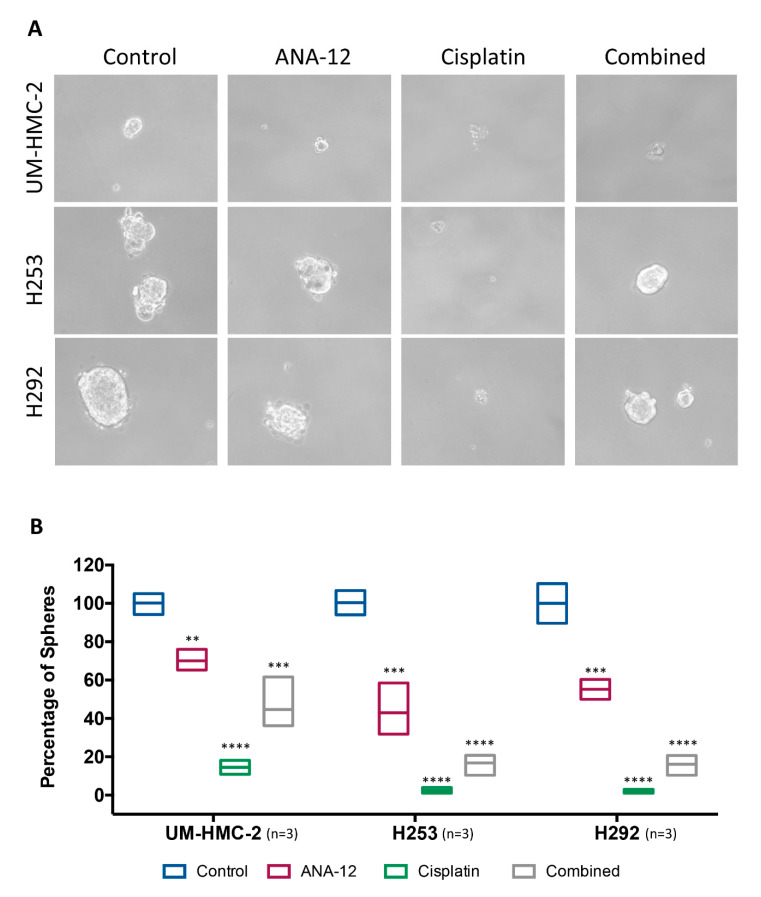
(**A**) Representative images of tumour spheroid under phase contrast microscopy. Note that cells under Cisplatin treatment have no ability to form well-developed spheres, however, in a combined group, this ability is recovered. (**B**) Quantitative analysis revealed that all treatments significantly reduced the number of spheres, however, it is possible to notice an increase in combined treatment compared to isolated Cisplatin (** *p* < 0.01; *** *p* < 0.001; **** *p* < 0.0001).

The tumour spheroid assay demonstrated that ANA-12 was efficient in disrupting the CSC population. The number of spheroids was significantly reduced, following TrkB inhibition in all MEC cell lines (Figure 6A,B). Surprisingly, in the present study, Cisplatin significantly disrupted the capacity to form spheroids in all MEC cell lines, this was in contrast to previous results that demonstrated a more limited effect [10]. An insignificant number of spheroids were formed, following Cisplatin administration, and they had a looser morphology compared to control spheroids (Figure 6A,B). Interestingly, but disturbingly, the combination of drugs allowed the recovery of colony formation by all MEC cell lines, under low-attachment conditions, suggesting that early TrkB inhibition protected the cells from the effects of Cisplatin. This phenomenon was not only seen in the recovery of the number of spheroids, but the morphology of the spheroids was more similar to the control group, with a more compact mass and clear edges (Figure 6A).

## 4. Discussion

SGC incidence will increase more than 55% in the next 22 years [1], highlighting the need to increase our comprehension of this life-threatening cancer. Currently, surgical approaches, such as parotidectomy or maxillectomy, remain the best options for achieving SGC disease control, however, they are associated with significant morbidity. Drug-based therapies are less promising, as effective drugs are currently not available, and unfortunately the majority of patients with metastatic SGC that require systemic treatment will succumb to their disease. Recently, the global paradigm for cancer treatment switched from nonspecific conventional drugs to highly selective targeted inhibition of signaling pathways involved with tumour acquisition or progression, taking into account individual molecular signatures [41]. This switch progressively encompasses different types of tumours such as breast, lung and prostate cancer, yet many cancers, including MEC, are still treated with outdated therapies. Our recent research was directed at further elucidating processes involved with MEC initiation, progression and resistance to conventional therapy, aimed at contributing to a better understanding of this tumour, and hopefully supporting the development of new treatments [11,41,42,43]. Our present study focused on the role of BDNF/TrkB in MEC progression, with the aim of understanding the effects of TrkB inhibition on key cellular events associated with disease relapse and metastasis. Our results initially suggested a promising effect of ANA-12 in impairing MEC cell migration and invasion in vitro, however, CSC accumulation following ANA-12 and Cisplatin combination treatment, represents an important limiting factor.

BDNF was initially identified in 1982, by Barde et al., as a cell survival-promoting factor for embryonic sensory neurons [23]. Currently, it is known that BDNF is the most abundant growth factor in the brain and plays a number of crucial roles, including regulation of dendritic cells, synaptic plasticity and hypothalamic metabolic function [24]. Non-neural tissues also express both BDNF and TrkB, suggesting that the diverse effect of this pathway is not restricted to the central and peripheral nervous system. Few studies investigated the basal levels of BDNF and TrkB in normal salivary gland tissue. However, Mandel et al. (2009) identified pro- and mature-BDNF in human saliva [44] while Saruta et al. (2012) demonstrated that BDNF was consistently expressed by ductal and serous cells of human submandibular glands [26]. We identified, through immunohistochemistry, that BDNF and phosphorylated TrkB are expressed in the ductal cells of the parotid gland and minor salivary glands. Interestingly, Batsakis (1980) hypothesized that MEC originated from stem cell progenitors located in proximity to the excretory duct [45]. Expression of BDNF/TrkB in normal ductal cells raises the question as to whether this pathway is constantly activated in normal ductal cells and remains activated in MEC, or whether these proteins are aberrantly activated and associated with a more aggressive phenotype. We observed a higher percentage of pTrkB-positive cells in MEC compared to normal salivary gland tissue, and a tendency of higher levels in high-grade cases, suggesting a possible association with aggressiveness. Interestingly, variable expression among cell lines evaluated was noted, but the high-grade MEC cell line demonstrated the highest TrkB mRNA levels, corroborating this hypothesis. This result was expected, as growth factors are known to be deregulated and overexpressed in many malignant tumours. Yet, the signaling pathways driven by growth factors, including BDNF/TrkB, are associated with many physiological conditions, and for this reason, we thought it would be important to compare protein expression of MEC with NSG. Our immunohistochemistry studies allowed us to determine the exact cellular localization of the TrkB protein and also focus on its expression in malignant MEC cells, rather than other stromal components, such as endothelial or neural cells present in the tissue samples. We detected phosphorylated TrkB expression in the cytoplasm of MEC cells, which is compatible with the receptor internalization, following phosphorylation in its active state. Increased expression of TrkB was noted in high-grade tumours, while cells involved with perineural invasion also expressed the protein, suggesting that TrkB activation might indeed be a feature associated with MEC aggressiveness. The mRNA results also corroborate this hypothesis, which was further endorsed by our functional in vitro assays, as TrkB inhibition significantly impaired oncogenic outcomes, such as cell proliferation, migration and survival. Differences in TrkB immunohistochemical levels among MEC grades did not achieve significance, but this was probably due to the small number of high-grade cases studied. The sample size represents a limitation of this study and further investigations with a more representative sample might uncover an association of TrkB with aggressive outcomes in MEC. We previously showed that BDNF or TrkB could play a role as biomarkers for reduced survival in other malignancies, such as oral squamous cell carcinoma [46] and melanoma [47], and this was further demonstrated in other adenocarcinomas, such as breast [48] and lung [49].

To further elucidate the role of the BDNF/TrkB pathway in MEC, we compared the effects of ANA-12 or cisplatin alone and in combination, on in vitro cell behaviour. Despite the lack of robust scientific evidence concerning the effects of Cisplatin on salivary gland cancers, this drug remains the most common chemotherapeutic agent used worldwide for this type of malignancy, and was thus included for comparison purposes. The combination of ANA-12 and Cisplatin was used as an approach to overcome the intrinsic resistance to cisplatin that MEC cells might harbour. Chemotherapeutic regimens using more than one drug at the same time can bring significant advantages. Bozic et al. [50], for example, determined through a mathematical approach, that the chances of achieving disease control in cutaneous melanoma presenting with 8 metastatic lesions was 0%, when only one chemotherapeutic agent was used, but the rates increased up to 88% when two drugs with different targets were combined [50]. ANA-12 exerts its main anti-neoplastic effects through TrkB inhibition [51], while Cisplatin interferes with DNA repair mechanisms, triggering DNA damage and cell apoptosis [31]. Our hypothesis was that, as TrkB activation is associated with increased resistance to Cisplatin in other cancers [52], ANA-12 would be effective as a sensitizing agent and would enhance the effects of Cisplatin. Lee et al. [52] revealed that endogenous BDNF can trigger expression of the drug-resistant protein MDR1, in head and neck squamous cell carcinoma [52]. We were unable to detect significant differences in MEC cell behaviour between the groups treated with Cisplatin alone or in combination with ANA-12. Multiple pathways can trigger intrinsic drug resistance and not all cell types will have similar molecular signatures. NFkB activation and histone acetylation were previously identified as regulators of intrinsic and adaptive resistance in MEC [10,11]. Our results suggest that the BDNF/TrkB pathway is not associated with these mechanisms and does not represent a promising target to increase Cisplatin sensitivity in MEC cells.

Analyzing the results from treatment with ANA-12 alone, allowed us to conclude that other oncogenic outcomes such as cell migration, invasion and survival, have a TrkB dependent mechanism. Previous studies demonstrated that TrkB inhibition can suppress epithelial–mesenchymal transition by modulating cell adhesion molecules and proteolytic enzymes, thus influencing a cell’s ability to invade the extracellular environment [53]. E-cadherin upregulation and the downregulation of N-cadherin and vimentin was noted following TrkB inhibition, which also reduced the levels of important degrading proteins, such as matrix metalloproteinase (MMP) 2 and MMP9 [53]. Our data demonstrated that ANA-12 reduced the invasive capacity of MEC cells, corroborating previous findings. Other studies demonstrated that BDNF/TrkB activation can regulate malignant cell resistance anoikis, the programmed-cell death fate of adherent cells, in response to inappropriate cell/ECM interactions that acts as a preventive mechanism for metastasis [24]. Taken together, these data support this pathway, which plays a crucial role in tumour progression and metastasis. A limitation of our study might be our inability to demonstrate the effect of ANA-12 on TrkB protein expression in MEC cells. The original effects described by Cazorla et al. (2011) [30] was recently confirmed in malignant cells [51], validating the inhibition of TrkB protein by ANA-12 in cancer cells.

The importance of CSC in tumour development and relapse is increasingly debated. Nguyen et al. [54] defined CSC as the sub-population of malignant cells that show cancer-propagating ability (can generate all the other cells within the tumour bulk), and suggested they must be eradicated to achieve disease control or cure [54]. Cell proliferation, migration, invasion, survival and apoptosis are all extremely important targets. However, if new drugs are not effective in eliminating the highly resilient and tumourigenic CSC population, patients might experience disease relapse. The tumour spheroid assay used in our study to evaluate CSC, can also be used to assess the capacity of drugs to achieve disease control. Colonies grown under low-attachment conditions express stem cell markers at significantly higher levels than cells grown in monolayer [37]. The effect of ANA-12 alone on the MEC CSC population showed that, on average, TrkB inhibition reduced the percentage of CSC in UM-HMC-2, H253 and H292 cells, by 30%, 57%, and 45%, respectively. All of our results achieved significance and suggest that TrkB inhibition can disrupt the CSC population. Future studies should be developed to determine whether the MEC CSC population, sorted through flow cytometry using ALDH and CD44 markers, show increased expression of TrkB, compared to a non-CSC population. Based on the effects of ANA-12 that we demonstrated, we expect this pathway to be associated to some extent with the MEC CSC phenotype. Yin et al. [54] found that in triple-negative breast cancer, TrkB+ CSC play a key role in post-chemotherapy disease relapse, corroborating our findings and suggesting that in some adenocarcinomas, TrkB inhibition could disrupt this cell sub-population. However, it is important to stress that CSC survival is based on a complex mechanism resulting from the interaction of many intrinsic and extrinsic factors. The combination of ANA-12 and cisplatin demonstrated that if ANA-12 was administered before cisplatin, MEC cells regained the stemness capacity lost, following treatment with cisplatin alone. This was contrary to our expectations of a chemosensitisation therapy. Normal stem cell behaviour is controlled by external signals and CSC are thought to respond in a similar way [55]. Moreover, malignant cells are known to have rapid and complex adaptive skills. TrkB does not appear to represent a vital pathway for MEC CSC survival, and a preliminary stress produced by ANA-12 might be triggering an intrinsic response that can induce Cisplatin resistance, such as activation of pro-survival and anti-apoptotic factors. Previous studies with MEC cell lines demonstrated a promising CSC sensitization therapy, by targeting other molecular events such as histone deacetylase inhibition (Vorinostat) [10] and NFkB inhibition (Emetine) [11]; this was not observed with TrkB inhibition.

We believe that these findings endorse the current concept of personalized cancer therapy. While TrkB is associated with CSC survival in breast cancer, MEC CSC are not dependent on this pathway. Thus, specific drugs for each type of cancer and each patient are needed, according to the molecular signature of the tumour. In vitro studies such as ours are vital in identifying how specific outcomes vary between cancer types and in determining which drugs are more promising for further pre-clinical and clinical trials.

## 5. Conclusions

The BDNF/TrkB pathway appears to be associated with aggressive cell behaviour in MEC. TrkB inhibition delayed migration and reduced invasion and survival of MEC cells in vitro. Unfortunately, combined treatment with ANA-12 and Cisplatin had a lesser effect on the decreasing CSC number, compared to Cisplatin alone, and this represents an important limiting factor.

## Supplementary Materials

The following are available online at https://www.mdpi.com/2227-9059/8/12/531/s1, Figure S1. MEC cell lines are derived from distinctive sites and original tumours had different grades. Yet, on cell culture, all cell types presented similar morphology under (A) phase-contrast microscopy (cobblestone epithelial-like) and (B) transmission electron microscopy (presence of intra-cytoplasmic vacuoles); Figure S2. (A) Representative images of the wound area of the UM-HMC-2 cell line during the wound healing assay. Note that at 48 h, only the control group presented a complete wound closure. (B) Representative images of the wound area of the H253 cell line, during the wound healing assay. Note that at 32 h, only the control group presented a complete wound closure; Figure S3. (A) Representative images of the MEC cell lines colonies and tumour spheroid. Note the disparities in size and morphology of colonies and tumour spheroid. Cells with highly compact chromatin (arrows) and abnormal mitotic activity (asterisks) could be noted. (B) Raw number of colonies differed between MEC cell lines, with the primary lung cell line (H292) presenting the highest value. (C) Raw number of tumour spheroid also differed, with high-grade MEC cell line (H253), which presented the greatest number of spheres.
