# Supplementary figures and images for: TrkB-Targeted Therapy for Mucoepidermoid Carcinoma
Source: Biomedicines. 2020 Nov 24;8(12):531. doi: 10.3390/biomedicines8120531 (PMC7759804; doi:10.3390/biomedicines8120531)

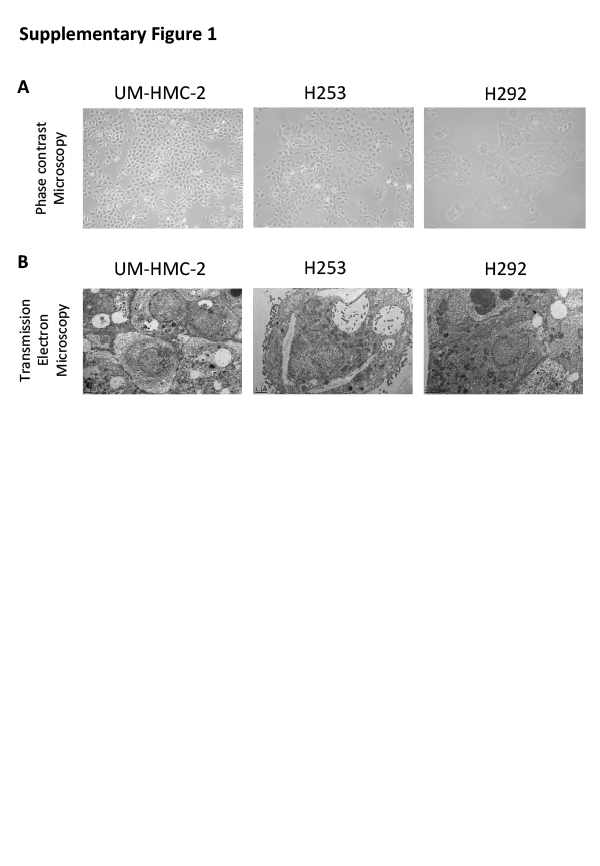

Supplement: Supplementary file 1 [file biomedicines-08-00531-s001.zip › Suppl Figure 1.tif]

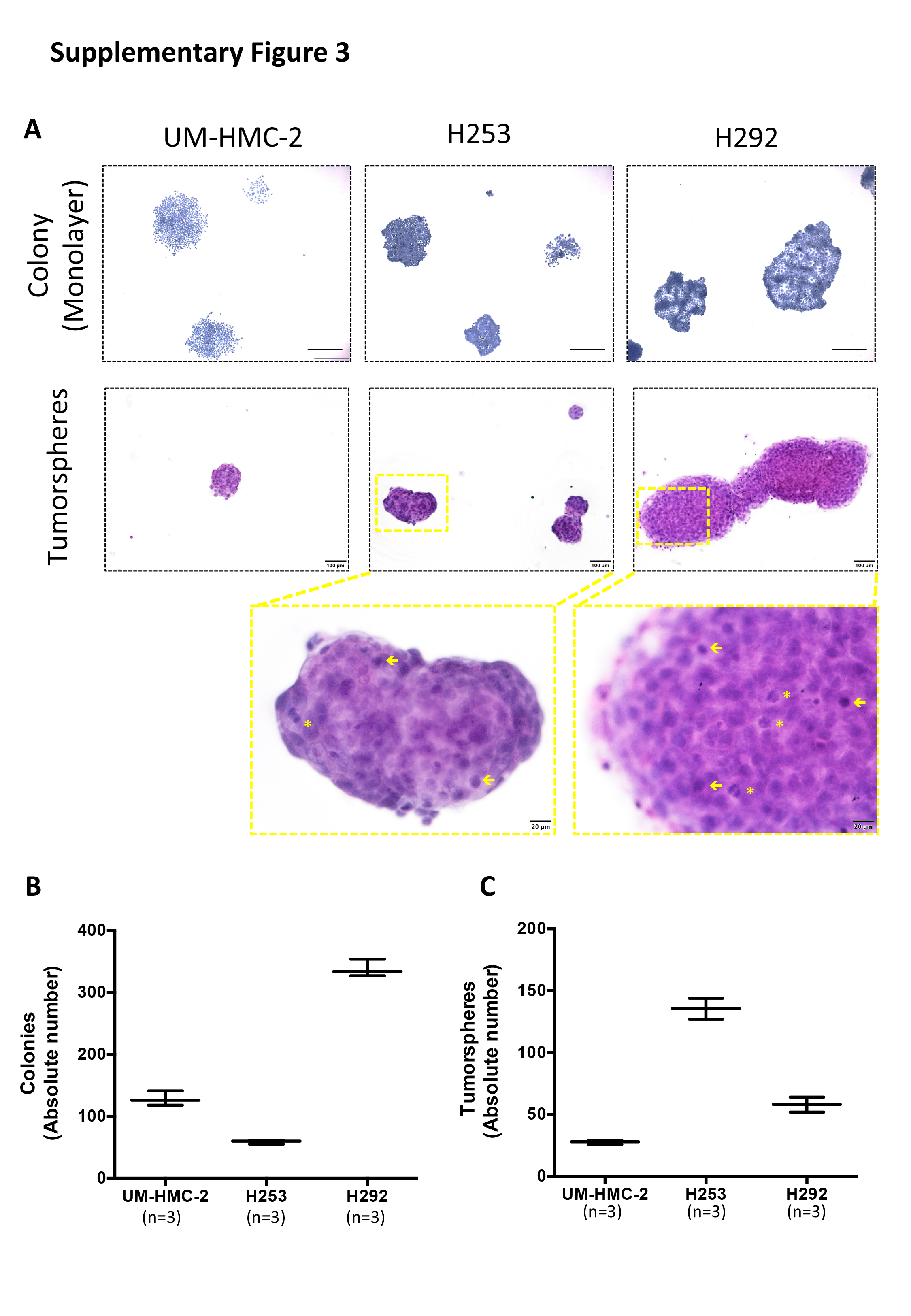

Supplement: Supplementary file 1 [file biomedicines-08-00531-s001.zip › Supp Figure 3.tif]

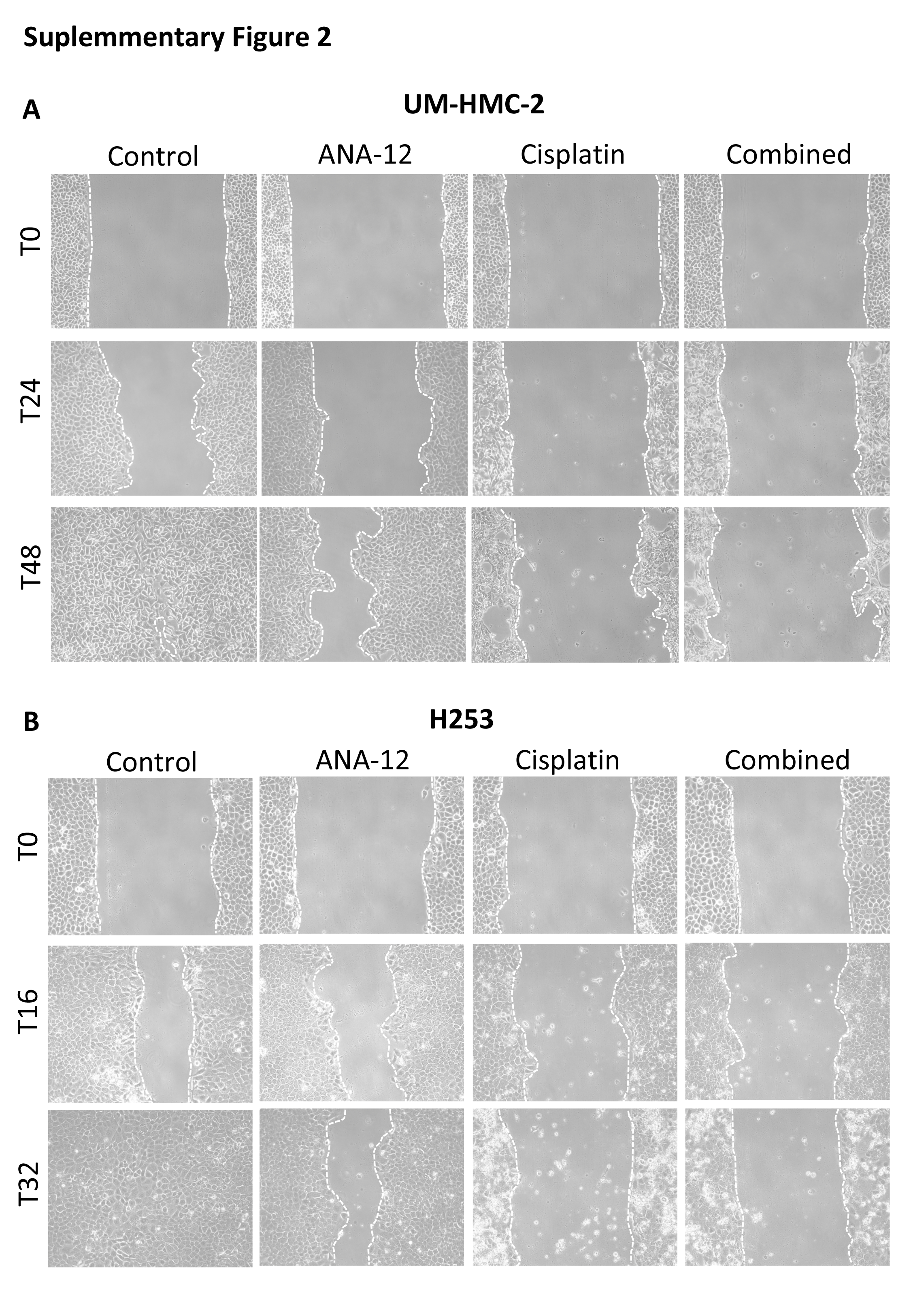

Supplement: Supplementary file 1 [file biomedicines-08-00531-s001.zip › Supp Figure 2.tif]
